# Programming a topologically constrained DNA nanostructure into a sensor

**DOI:** 10.1038/ncomms12074

**Published:** 2016-06-23

**Authors:** Meng Liu, Qiang Zhang, Zhongping Li, Jimmy Gu, John D. Brennan, Yingfu Li

**Affiliations:** 1 Department of Biochemistry and Biomedical Sciences, McMaster University, 1280 Main Street West, Hamilton, Ontario, Canada L8S 4K1; 2 Biointerfaces Institute, McMaster University, 1280 Main Street West, Hamilton, Ontario, Canada L8S 4O3

## Abstract

Many rationally engineered DNA nanostructures use mechanically interlocked topologies to connect individual DNA components, and their physical connectivity is achieved through the formation of a strong linking duplex. The existence of such a structural element also poses a significant topological constraint on functions of component rings. Herein, we hypothesize and confirm that DNA catenanes with a strong linking duplex prevent component rings from acting as the template for rolling circle amplification (RCA). However, by using an RNA-containing DNA [2] catenane with a strong linking duplex, we show that a stimuli-responsive RNA-cleaving DNAzyme can linearize one component ring, and thus enable RCA, producing an ultra-sensitive biosensing system. As an example, a DNA catenane biosensor is engineered to detect the model bacterial pathogen *Escherichia coli* through binding of a secreted protein, with a detection limit of 10 cells ml^−1^, thus establishing a new platform for further applications of mechanically interlocked DNA nanostructures.

## Abstract (web summary)

DNA nanostructures with interlocked topologies will tend to display different behaviour to the linear counterparts. Here, the authors show a DNA catenane that is inactive for rolling circle amplification but is activated upon cleavage of one ring, and exploit this for the development of a biosensing system.

## Graphical abstract

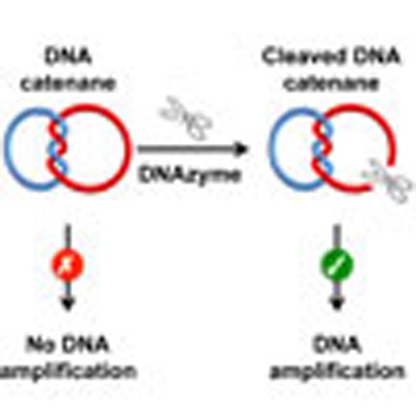

DNA is not only important in biological systems as genetic material, it has also become a key player in synthetic biology. DNA can be engineered into catalysts (DNAzymes) and molecular receptors (DNA aptamers), making DNA a functionally versatile polymer. DNA, as a highly programmable material based on predictable Watson–Crick base-pairing interactions, has become a valuable macromolecule for rational engineering of molecular machines for potential nanotechnological applications.

In recent years, tremendous progress has been made toward building DNA-based nanodevices with increasing structural complexity and functional capabilities^1^,^2^,^3^,^4^,^5^,^6^,^7^,^8^,^9^,^10^,^11^,^12^. One important feature of many reported DNA nanostructures, such as DNA Borromean rings^1^ and DNA catenanes^3^, is the use of mechanically interlocked topologies to connect individual DNA components. The mechanical interlocking between DNA strands can be easily achieved in the case of DNA through the formation of a linking duplex between partner rings before ring closure. The existence of a linking duplex is not only essential to the creation of a strong connectivity between partner rings but also necessary for the stability of these well-defined structures.

As we will show in this work, the linking-duplex feature also enables the use of topologically interlocked architectures, such as DNA catenanes, for the design of amplified biosensors for bioanalytical applications. The biosensing strategy is based on the following idea: the strong physical engagement of two mechanically interlocked single-stranded DNA rings in a DNA [2] catenane (termed D2C in this study for simplicity) with a strong linking duplex makes the component rings unsuitable as the template for rolling circle amplification (RCA), an isothermal DNA amplification technique^13^,^14^,^15^. However, when one of the component rings is engineered to be a substrate of a stimuli-responsive RNA-cleaving DNAzyme (RCD), the system can be programmed into a biosensor that is capable of reporting a target of interest in three sequential reactions: target-induced RNA cleavage, nucleolytic conversion of the cleavage product into a DNA primer, and DNA amplification via RCA. By this approach, we establish an amplified biosensing system that is capable of achieving ultra-sensitive detection of *Escherichia coli* (*E. coli*) at a concentration as low as 10 cells ml^−1^ without cell culture.

## Results

### Inability of a D2C with a strong linking duplex to undergo RCA

**Figure 1:**
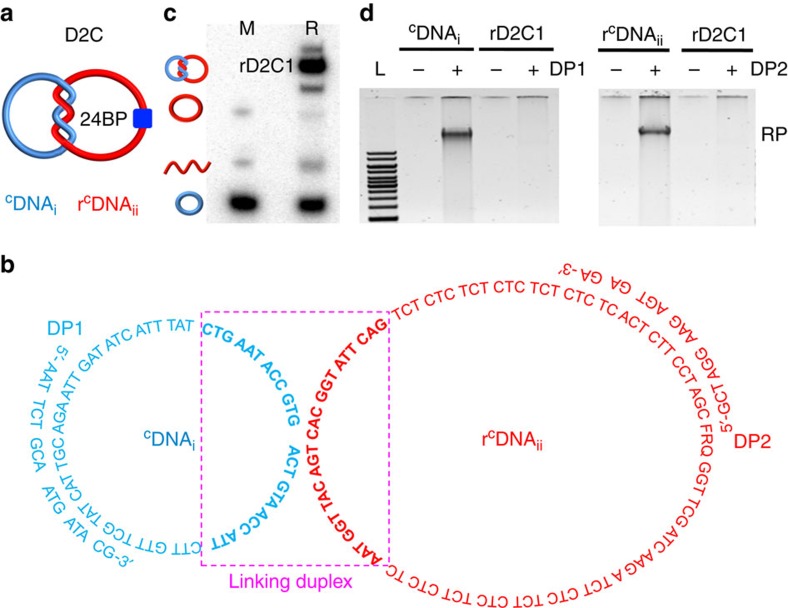
Inability of a D2C to undergo RCA. (**a**) Schematic illustration of a D2C made of ^C^DNA_i_ and r^C^DNA_ii_, with a linking duplex of 24 bp. (**b**) Sequences of rD2C1, DP1 and DP2. Boxed nucleotides represent the 24-bp linking duplex. F: fluorescein-dT; R, adenosine ribonucleotide; Q, dabcyl-dT. (**c**) Synthesis of rD2C1 by circularizing linear DNA_ii_ over ^C^DNA_i_ as the template. Lane M, markers made of ^L^DNA_ii_, ^C^DNA_i_ and r^C^DNA_ii_. Lane R: circularization mixture. (**d**) RCA reactions with gel-purified ^C^DNA_i_, r^C^DNA_ii_ and rD2C1 using DP1 and DP2 as primers. Lane L, DNA ladders ranging from 1 to 10 kbp; RP, RCA product.

The conceptual cornerstone of this work is the assumption that the components of a D2C with a strong linking duplex are unable to undergo RCA. To test this idea, we synthesized a D2C (Fig. 1a) consisting of two component single-stranded DNA rings, named ^C^DNA_i_ and r^C^DNA_ii_ (Fig. 1b; r stands for the single ribonucleotide, ribo-A, in the sequence of ^C^DNA_ii_). Briefly, the linear DNA r^L^DNA_ii_ was circularized into r^C^DNA_ii_ using ^C^DNA_i_ as the ligation template (sequences of all DNA species are provided in Supplementary Table 1). The resultant D2C, denoted rD2C1, contains a strong linking duplex of 24 bp, which translates into two helical turns (boxed nucleotides in Fig. 1b). The reaction yield of rD2C1 was determined to be 58% (Fig. 1c).

We then performed RCA reactions with gel-purified ^C^DNA_i_, r^C^DNA_ii_ and rD2C1. Agarose gel analysis indicated that RCA products were produced with ^C^DNA_i_ (using DP1 as the primer; Fig. 1d) and r^C^DNA_ii_ (using DP2 as the primer; Fig. 1d). In contrast, no RCA products were observed for rD2C1 using the same set of primers (Fig. 1d). This experiment shows that the topological constraint imposed by a strong linking duplex indeed prevents ϕ29 DNA polymerase (ϕ29DP) from replicating interlocked circular templates. In a control experiment, it was found that RCA was not inhibited when the linking duplex of the DNA catenane was made of 9 bp (Supplementary Fig. 1).

### Enabling RCA via cleavage of a component ring by a DNAzyme

The inability of ϕ29DP to carry out RCA with topologically constrained DNA catenanes provides a novel avenue to explore these intricate DNA assemblies for practical applications. Given our interest in DNAzymes and biosensing, we turned our attention next to the engineering of a highly unique biosensing system that takes advantage of topologically constrained DNA catenanes, DNAzymes and RCA.

**Figure 2:**
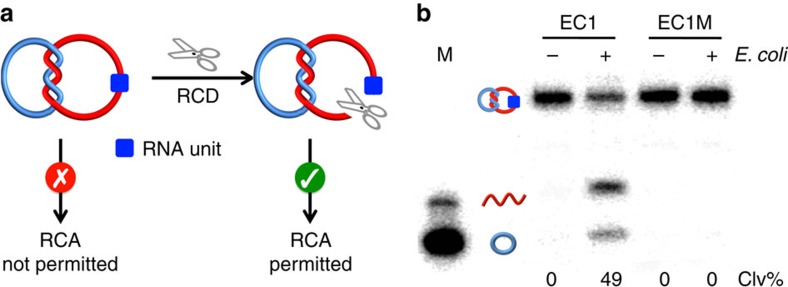
Cleavage of an RNA-containing D2C by an RCD. (**a**) Restoration of RCA compatibility of an rD2C using an RCD. (**b**) Cleavage of rD2C1 by EC1, an *E. coli*-responsive DNAzyme. Concentration of *E. coli*: 10^5^ cells ml^−1^. Reaction mixtures were analyzed by 10% denaturing PAGE. EC1M: a mutant EC1 that cannot be activated by *E. coli*. Both r^C^DNA_ii_ and ^C^DNA_i_ in rD2C1 were radioactively labelled with ^32^P to facilitate DNA visualization on the gel. Clv%: per cent cleavage.

The working principle of our biosensing system is shown in Fig. 2a. It uses an RCD to cleave the embedded RNA linkage within rD2C1 (note that the r^C^DNA_ii_ was designed to contain a single ribonucleotide; Fig. 1b). It is expected that the cleavage and linearization of r^C^DNA_ii_ by the RCD will release the topological constraint on the DNA assembly, which converts ^C^DNA_i_ into a suitable template for RCA.

In theory, our approach should be compatible with any RCD, making it a platform for detection of any species recognized by an allosteric DNAzyme. For this work, we employed EC1, which was previously isolated by us from a random-sequence DNA pool using *in vitro* selection for specific detection of *E. coli*, a model bacterial pathogen^16^,^17^. EC1 was found to be activated by a protein molecule secreted specifically by *E. coli* cells (illustrated in Supplementary Fig. 2). Therefore, the use of EC1 enables the detection of this pathogen. As illustrated in Fig. 2b, EC1 was indeed able to cleave the r^C^DNA_ii_ present in rD2C1 in an *E. coli*-dependent manner, resulting in ^C^DNA_i_ and a linear DNA_ii_. An inactive DNAzyme mutant, EC1M, was also tested as a control. No cleavage product was observed for EC1M, indicating that the cleavage reaction is highly dependent on the DNAzyme sequence. We also examined the cleavage activity at different reaction times and found that the cleavage activity reached a plateau in 1 h (Supplementary Fig. 3). Thus, this reaction time was used for the remaining experiments.

**Figure 3:**
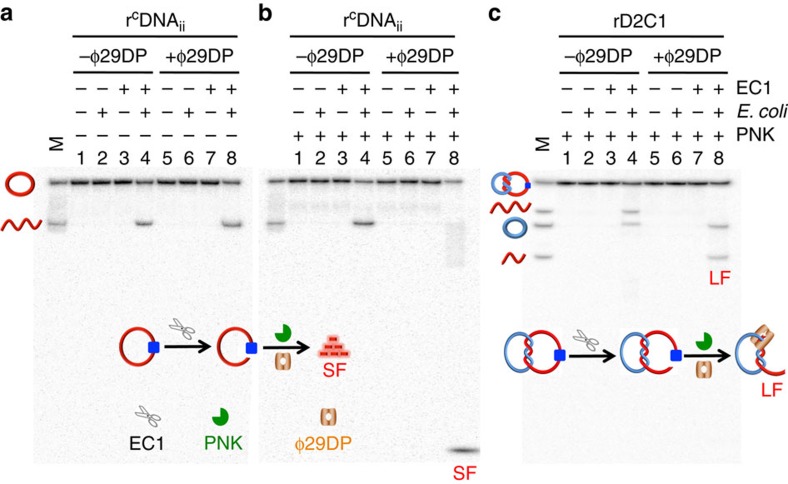
3′–5′ exonucleolytic activity of ϕ29DP on r^C^DNA_ii_ and rD2C1. Degradation of EC1-mediated cleavage product of r^C^DNA_ii_ (**a**,**b**) and rD2C1 (**c**) by ϕ29DP and PNK. Concentration of *E. coli*: 10^5^ cells ml^−1^. Reaction mixtures were analyzed by 20% denaturing PAGE. LF, large DNA fragment; SF, small DNA fragment. M lanes contain various DNA markers as indicated. r^C^DNA_ii_, both r^C^DNA_ii_ and ^C^DNA_i_ in rD2C1 were radioactively labelled with ^32^P to facilitate DNA visualization on the gel.

Upon demonstrating EC1-mediated cleavage of r^C^DNA_ii_ in rD2C1, we examined the use of the cleavage reaction mixture for initiating the RCA reaction with ϕ29DP. Because ϕ29DP has 3′–5′ exonucleolytic activity that can degrade single-stranded DNA from the 3′-end but does not digest double-stranded DNA^18^,^19^, we speculated that the system should not require an external primer, as ϕ29DP should be able to convert the linearized DNA_ii_ into a primer for RCA. To evaluate this hypothesis, we determined whether ϕ29DP could digest EC1-linerized DNA_ii_. From the data presented in Fig. 3a, it is clear that ϕ29DP could not digest linearized DNA_ii_ (comparing lanes 4 and 8).

There are two possible reasons for this finding. The first possibility is that ϕ29DP is incapable of digesting an RNA-terminated DNA molecule. However, testing with an RNA-terminated oligonucleotide ruled out this possibility (Supplementary Fig. 4). The second scenario is that ϕ29DP is not able to digest an RNA-terminated DNA molecule containing a 2′,3′-cyclic phosphate on the RNA moiety, which is a common product of RNA cleavage^20^. To test this, we treated the reaction mixture with T4 polynucleotide kinase (PNK), which is known to be capable of removing the terminal 2′,3′-cyclic phosphate in RNA^21^. As shown in Fig. 3b, treatment with PNK indeed facilitated the digestion of EC1-linearized rDNA_ii_, as evidenced by the accumulation of small cleavage fragments (labelled SF in lane 8).

We next investigated the combined action of ϕ29DP and PNK on EC1-linearized rDNA_ii_ within rD2C1. As shown in Fig. 3c, ϕ29DP degraded complexed DNA_ii_ to a product of ∼60 nt (labelled LF, representing long fragment; lane 8). We also conducted the digestion assays using different incubation times (Supplementary Fig. 5). The progressive accumulation of LF and disappearance of the cleaved rDNA_ii_ was observed. These experiments allow us to conclude that the combination of ϕ29DP and PNK can remove the single-stranded fragment at the 3′-end of the EC1-linearized DNA_ii_ from rD2C1. It is expected that the trimmed DNA_ii_ can now function as a primer to initiate RCA over the complexed ^C^DNA_i_ template.

**Figure 4:**
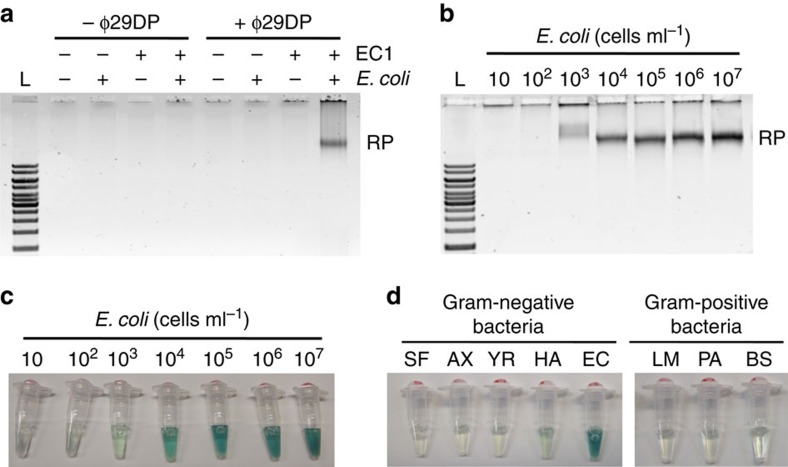
*E. coli*-dependent RCA reaction. (**a**) RCA reactions of rD2C1 in the presence of *E. coli* (10^5^ cells ml^−1^) analyzed using 0.6% agarose gel electrophoresis. Note every reaction also contained PNK and dNTPs. L, DNA ladders ranging from 1 to 10 kbp; RP, RCA product. (**b**) Determination of detection sensitivity through analysis of RP using 0.6% agarose gel electrophoresis. (**c**) Determination of detection sensitivity via the colourimetric assay enabled by PW17 peroxidase DNAzyme. (**d**) Analysis of assay specificity using the colourimetric assay. The gram-negative bacteria used were *Serratia fonticola* (SF), *Achromobacter xylosoxidans* (AX), *Yersinia ruckeri* (YR) and *Hafnia alvei* (HA). The gram-positive bacteria used were *Leuconostoc mesenteroides* (LM) and *Pediococcus acidilactici* (PA).

To verify the point above, we carried out the RCA reaction with the rD2C1 assembly. The reaction was performed in two sequential steps: activation of EC1 by *E. coli* and PNK treatment, followed by the addition of ϕ29DP and dNTPs. As expected, RCA products were indeed observed following this procedure (the last lane of Fig. 4a; the other lanes represent various controls). The RCA products were further analyzed through partial digestion with *Eco*RV (Supplementary Fig. 6) as the sequence of ^C^DNA_i_ was designed to contain a recognition sequence for this restriction enzyme. The appearance of the expected characteristic DNA banding pattern on the gel, which consists of monomeric, dimeric and other higher-ordered DNA amplicons, verified that the RCA products indeed contained the correct repetitive sequences.

### Quantitative detection of *E. coli* using the DNA catenane sensor

We then investigated the feasibility of performing quantitative analysis using the DNA catenane sensor. Samples containing at *E. coli* 10–10^7 ^cells ml^−1^ were assessed for RCA amplified detection using a gel-staining method. By this method, we were able to detect as low as 10^3^ cells ml^−1^ (Fig. 4b). Although gel-based RP analysis can perform quantitative detection of *E. coli*, the procedure is extremely inconvenient. To overcome this issue, we developed a colorimetric assay by modifying the sequence of ^C^DNA_i_ (the new sequence is named ^C^DNA_i_CD) such that the RCA product contained a repetitive sequence of PW17, a peroxidase-like DNAzyme capable of generating a colourimetric signal^22^,^23^,^24^,^25^,^26^. In the presence of hemin, PW17 catalyzes the H_2_O_2_-mediated oxidation of 2,2′-azino-bis(3-ethylbenzthiazoline-6-sulphonic acid) (ABTS) into a coloured product. As shown in Fig. 4c, this colourimetric method was indeed able to detect *E. coli* in a concentration-dependent manner and registered a detection sensitivity of 10^3^ cells ml^−1^, similar to what was observed with the gel-based method.

We also evaluated the bacterial detection specificity using the colourimetric assay. We selected four other Gram-negative and three Gram-positive bacteria that were previously tested for EC1-based detection. It was observed that none of these bacteria were able to produce a positive signal, indicating that the rD2C1/EC1 system retained the high recognition specificity for *E. coli* (Fig. 4d). To further evaluate the specificity, we checked the potential influence of small RNAs on *E. coli* detection due to the fact that small RNAs (for example, microRNA) are suitable primers for RCA. For this experiment, we used the total small RNAs extracted from breast cancer cell line MCF-7. Agarose gel and colourimetric results indicated that the small RNAs were not able to induce the RCA reaction (Supplementary Fig. 7). This high specificity is attributed to the unique topologically constrained structure of the DNA catenane.

### Enhancing detection sensitivity using hyperbranched RCA

**Figure 5:**
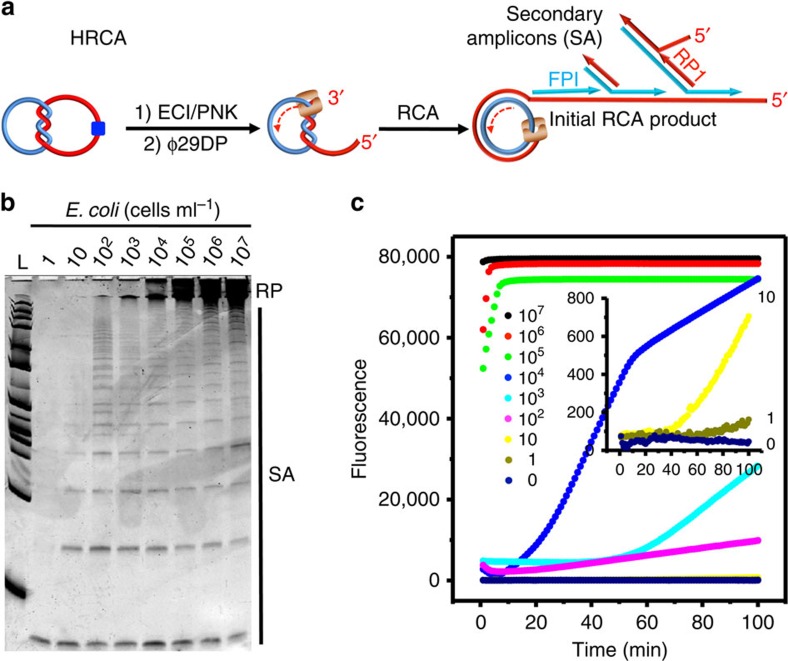
*E. coli*-dependent HRCA reaction. (**a**) Schematic illustration of HRCA. FP1, forward primer; RP1, reverse primer. (**b**) Denaturing PAGE analysis of HRCA products. RP, RCA products; SA, secondary amplicons produced from the initial RCA products. (**c**) Real-time monitoring of HRCA reactions at various *E. coli* concentrations (cells ml^−1^).

Finally we examined the possibility of performing a double-primed hyperbranched RCA (HRCA)^27^ reaction with the rD2C1/EC1 system to further increase the detection sensitivity. In HRCA (Fig. 5a), multiple priming events can be continuously initiated by a forward primer (FP1) and a reverse primer (RP1) as the original RCA product strand elongates, resulting in an exponential amplification^28^.

We found that HRCA was indeed functional with the rD2C1/EC1 system: as shown in Fig. 5b, in addition to the observation of the RCA products on denaturing polyacrylamide gel electrophoresis (PAGE), a series of shorter DNA molecules were also produced, representing various secondary amplicons produced from the primary amplicons (that is, initial RCA products). The HRCA reactions, in response to varying concentrations of *E. coli*, were also monitored in real time through the use of EvaGreen, a DNA-binding dye (Fig. 5c). We found that this method exhibited much enhanced detection sensitivity, as it was able to detect *E. coli* at a concentration of as low as 10 cells ml^−1^ without cell culture.

It is interesting to note that RCA reactions with ϕ29DP remain functional even in the presence of 50% human blood (Supplementary Fig. 8). In comparison, PCR with Taq DNA polymerase was completely inhibited by less than 0.2% human blood^29^,^30^,^31^. To demonstrate the performance of the assay when using more complex samples, we spiked whole blood with *E. coli* and demonstrated that under these conditions the DNA catenane sensor was still able to detect *E. coli* at a concentration of 10 cells ml^−1^ (Supplementary Fig. 9). This observation is consistent with a recent report where EC1 was used to detect *E. coli* cells in human blood^32^.

The ability of the DNA catenane sensor to distinguish between live and dead *E. coli* cells was also investigated. For this experiment, we used lysozyme to kill *E. coli* (10^5 ^cells ml^−1^) and compared the signal responses of the catenane sensor in the presence of live and dead bacteria using HRCA. Negligible signal was observed with dead cells while a high activity was seen with live *E. coli* cells (Supplementary Fig. 10).

We also performed an *E. coli* detection experiment with an enzyme-linked immunosorbent assay (ELISA) using a commercial ELISA kit designed to detect *E. coli* host cell protein (Supplementary Fig. 11). It was found that the ELISA method was able to detect *E. coli* at a concentration of 10^3^ cells ml^−1^. Therefore, our amplified DNA catenane sensor offers a detection sensitivity that is 100 times better than the ELISA method, while the test time for both methods are similar (∼3 h; see Supplementary Table 2 for additional information).

## Discussion

We have shown for the first time that mechanically interlocked DNA [2] catenanes with a strong linking duplex impose a significant topological constraint on their component DNA rings, making them unsuitable as the template for RCA. We have further demonstrated that such DNA nanostructures can be uniquely exploited for the design of a biosensing system where the elimination of the topological engagement, achieved simply through the cleavage of one interlocked DNA ring in an analyte-dependent manner, frees up the other ring for the RCA reaction. As an example, we have produced one of the two interlocked DNA rings to contain a RNA linkage so that an RCD can be used to cleave one interlocked ring. Through the use of an RCD whose activity is specifically triggered by a secreted protein in *E. coli*, we have shown that the featured biosensing system is capable of achieving ultra-sensitive detection of this bacterial pathogen.

The biosensing system featured here offers some distinct advantages over existing detection methods for *E. coli*, such as cell culturing, PCR and ELISA (see Supplementary Table 2 for additional information). The use of RCA and HRCA for signal amplification makes this system extremely sensitive for bacterial detection, which can achieve the detection of as low as 10 cells ml^−1^ without a cell-culturing step. The assay is also more compatible with point-of-care or field applications because RCA is an isothermal process and there is no need for DNA extraction (as in the case of PCR). In addition, the system functions well with biological samples (no interferences from small RNAs and compatibility with blood samples).

It is conceivable that the same design can be extended to other RCDs, DNA-cleaving DNAzymes, as well as ribozymes and protein enzymes that have DNA or RNA-cleaving activities. Although rolling circle amplification was exploited to achieve signal amplification in this study, it should be feasible to take advantage of other signal amplification strategies, such as the DNAzyme cross-amplification system developed by Levy and Ellington that does not need a DNA polymerase^33^. We envision that the concept presented in this study should open up new opportunities for exploring mechanically interlocked DNA architectures for many potential applications in chemical biology, medical diagnostics, and environmental monitoring.

## Methods

### Preparation of rD2C1

A total of 100 pmol of r^L^DNA_ii_ was first labelled with γ-[^32^P]ATP at the 5′ end using T4 PNK according to the manufacturer's protocol. To ensure that all DNA molecules contain the 5′ phosphate required for the subsequent ligation reaction, PNK-mediated end-labelling solution containing 5′-^32^P labelled r^L^DNA_ii_ was further incubated with 2 mM non-radioactive ATP at 37 °C for 30 min. Then 120 pmol of ^C^DNA_i_, prepared using the procedure detailed in Supplementary Methods, was added and heated to 90 °C for 30 s. After cooling to room temperature and leaving the solution for 15 min, 10 μl of 10 × T4 DNA ligase buffer and 10 U T4 DNA ligase were added (total 100 μl) and incubated at room temperature for 2 h. The obtained rD2C1 molecules were concentrated by standard ethanol precipitation and purified by 10% dPAGE.

### *E. coli*-dependent RCA reaction

A single colony of *E. coli* K12 freshly grown on Luria broth (LB) agar plate was taken and used to inoculate 2 ml of LB. After shaking at 37 °C for 14 h at 250 r.p.m., the bacterial culture was serially diluted in 10-fold intervals. Overall, 100 μl of each diluted solution was plated onto LB agar plate (done in triplicate) and cultured at 37 °C for 15 h to obtain the cell counts. Colonies in each plate were counted; the average number of colonies from the three plates was taken as the number of *E. coli* cells for this dilution. This number was then used to calculate the number of cells for the other dilutions. A total of 500 μl of each dilution was centrifuged at 13,000*g* for 20 min at 4 °C and re-suspended in 100 μl of 1 × RB (50 mM HEPES, 150 mM NaCl, 15 mM MgCl_2_, pH 7.5). After being frozen at −20 °C, *E. coli* cells were sonicated for 1 min and put on the ice for 5 min. This process was repeated three times. Then the cell suspension containing different numbers of *E. coli* cells were centrifuged at 13,000*g* for 10 min at 4 °C. The obtained crude intracellular mixture produced by *E. coli* cells (CIM-EC) in the supernatant was used for the following experiment.

A cleavage reaction mixture containing 5 μl of CIM-EC, 1 μl of rD2C1 (5 μM), 4 μl of EC1 (50 μM) and 10 μl of 2 × RB was incubated at room temperature for 60 min. Then 1 μl of PNK (10 U μl^−1^) was added and incubated at 37 °C for 30 min. The RCA reaction was initiated by the addition of 1 μl of ϕ29DP (10 U μl^−1^), 1 μl of dNTPs (50 mM), 5 μl of 10 × RCA reaction buffer and 22 μl of water. The reaction mixtures were incubated at 30 °C for 60 min before heating at 90 °C for 5 min. The resultant RCA products were analyzed by 0.6% agarose gel electrophoresis.

### Colourimetric detection

rD2C1 used for the colourimetric detection of *E. coli* was made of r^C^DNA_ii_ and ^C^DNA_i_CD, prepared using the procedure described in Supplementary Methods. After the cleavage reaction described above, 1 μl of PNK (10 U μl^−1^) was added and incubated at 37 °C for 30 min. The RCA reaction was then initiated by the addition of 1 μl of ϕ29DP (10 U μl^−1^), 1 μl of dNTPs (50 mM), 2 μl of hemin (100 μM), 5 μl of 10 × RCA reaction buffer and 20 μl of water. The reaction mixtures were incubated at 30 °C for 60 min before heating at 65 °C for 20 min. After cooling to room temperature, 2 μl of ABTS (50 mM) and 1 μl of H_2_O_2_ (8.8 mM) were added, and the colourimetric result was recorded immediately using a digital camera.

### HRCA reaction

Following the cleavage reaction, 1 μl of PNK (10 U μl^−1^) was added and incubated at 37 °C for 30 min. The HRCA reaction was then initiated by the addition of 1 μl of ϕ29DP (10 U μl^−1^), 1 μl of dNTPs (50 mM), 1 μl of FP1 (50 μM), 1 μl of RP1 (50 μM), 5 μl of 10 × RCA reaction buffer, 2.5 μl of 20 × EvaGreen and 17.5 μl of water. These reactions were carried out in BioRad CFX96 qPCR system set to a constant temperature of 30 °C, and the fluorescence intensity was recorded in 1 min intervals.

### Other experiments

Details for the following experiments are provided in Supplementary Methods: preparation of r^L^DNA′_ii_ and r^C^DNA′_ii_, and rD2C1′; comparison of the cleavage activity of EC1 and EC1M in the presence of *E. coli*; degradation of rD2C1 by ϕ29DP in the presence of EC1, *E. coli* and PNK; cell culture and miRNA extraction; restriction digestion of RCA products; effect of blood on RCA reactions; detection of *E. coli* in blood samples; and detection of *E. coli* using an ELISA kit.

### Data availability

The authors declare that the data supporting the findings of this study are available within the article and its Supplementary Information files.

## Additional information

**How to cite this article:** Liu, M. *et al*. Programming a topologically constrained DNA nanostructure into a sensor. *Nat. Commun.* 7:12074 doi: 10.1038/ncomms12074 (2016).

## Supplementary Material

Supplementary Information Supplementary Figures 1-11, Supplementary Tables 1-2 and Supplementary Methods
