# Supplementary material for: Programming a topologically constrained DNA nanostructure into a sensor
Source: Nat Commun. 2016 Jun 23;7:12074. doi: 10.1038/ncomms12074 (PMC4931013; doi:10.1038/ncomms12074)
Supplement: Supplementary Information — Supplementary Figures 1-11, Supplementary Tables 1-2 and Supplementary Methods [file ncomms12074-s1.pdf]

Supplementary Figures

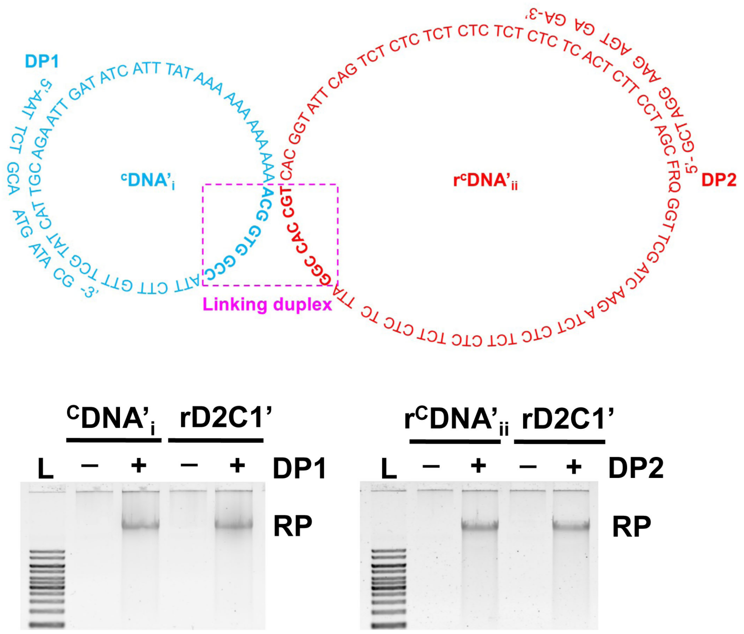

Supplementary Figure 1. RCA reactions with ^{C}DNA'$_{i}$, r^{C}DNA'$_{ii}$ and rD2C1' using DP1 and DP2 as primers. (a) Sequence of rD2C1'. It contains a linking duplex of 9 base pairs (boxed nucleotides); in comparison, rD2C1 has a linking duplex of 24 base-pairs (see Figure 1b of the main manuscript). F: fluorescein-dT; R: adenosine ribonucleotide; Q: dabcyl-dT. (b) and (c) RCA reactions using DP1 and DP2 as primers, respectively. RP: RCA product. Lane L: DNA ladders ranging from 1-10 kilo base-pairs.

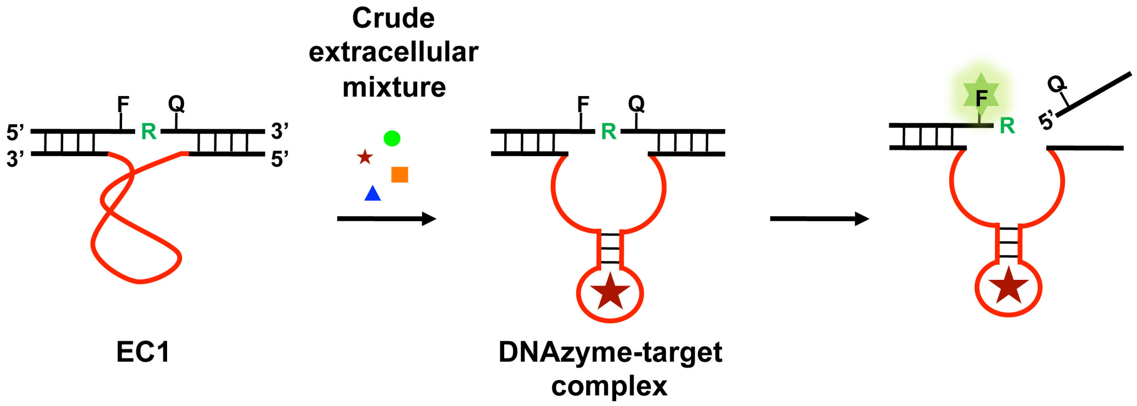

Supplementary Figure 2. Schematic illustration of the activity of EC1, an *E. coli*-responsive DNAzyme. EC1 cleaves a chimeric DNA/RNA substrate at a lone RNA linkage (green R) flanked by two nucleotides labeled with a fluorophore (F) and a quencher (Q), respectively, and more importantly, the cleavage activity of EC1 is dependent on an undeciphered protein molecule (represented by the red star) screted specifically by *E. coli*. The DNAzyme works simply by incubating EC1 with the crude extracellular mixture of *E. coli* as it contains the targeted protein molecule.

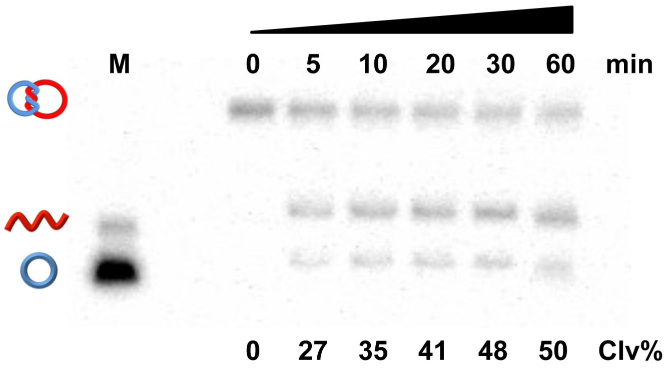

Supplementary Figure 3. 10% dPAGE analysis of cleaved products of rD2C1 generated upon incubation in the presence of EC1 and *E. coli* for different incubation times.

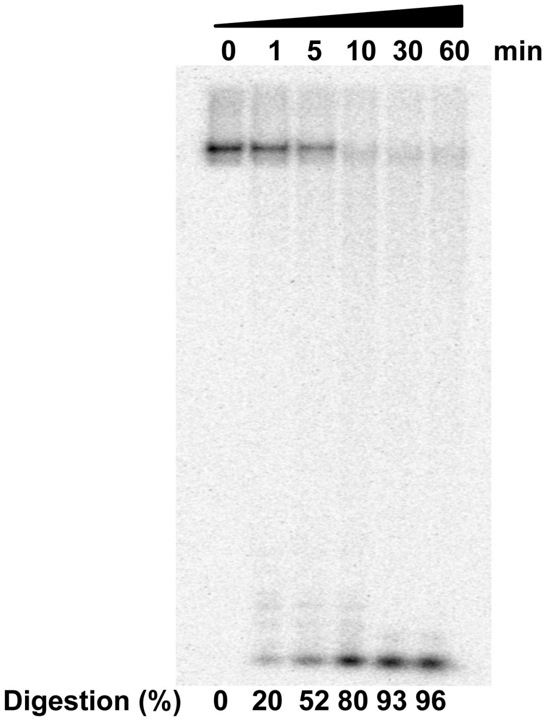

Supplementary Figure 4. Degradation of r^{L}DNA$_{ii}$ by ϕ29DP. All the reactions were carried out at 30°C in 20 μL of 1× RCA reaction buffer containing 250 nM ^{L}DNA$_{ii}$ and 5 U ϕ29DP. The degradation products were analyzed by 20% dPAGE.

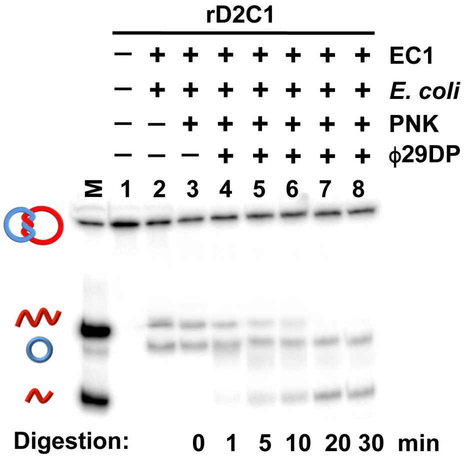

Supplementary Figure 5. Cleavage of rD2C1 by ϕ29DP at different digestion times in the absence or presence of EC1, *E. coli*, PNK or all components.

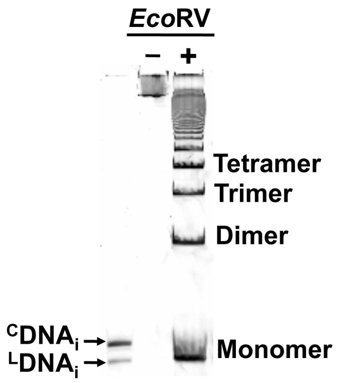

Supplementary Figure 6. Analysis of *Eco*RV-digested RCA products by 10% dPAGE.

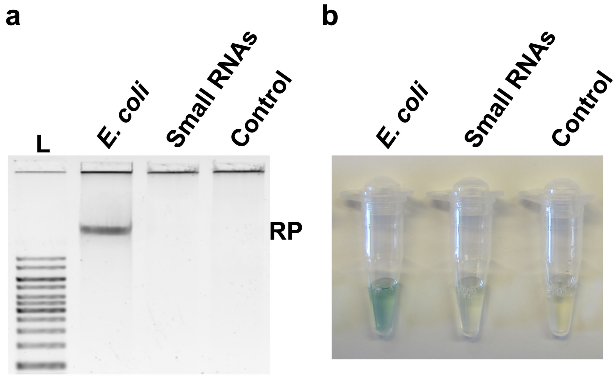

Supplementary Figure 7. RCA reactions of rD2C1 in the presence of *E. coli* (10^4 cells mL^{-1}) and small RNAs (5 ng, prepared from breast cancer cell line MCF-7) analyzed using (a) agarose gel electrophoresis and (b) colourimetric assay. RP: RCA product. L: DNA ladders ranging from 1-10 kilo base-pairs.

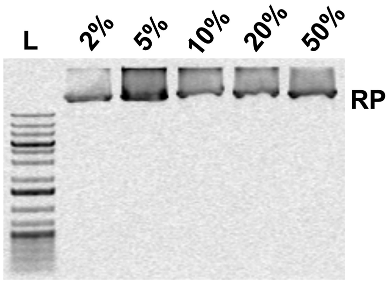

Supplementary Figure 8. Effect of blood on RCA reactions. RCA reactions of $^{C}DNA_{i}$ and DP1 were performed in the presence of 2, 5, 10, 20 and 50% human blood.

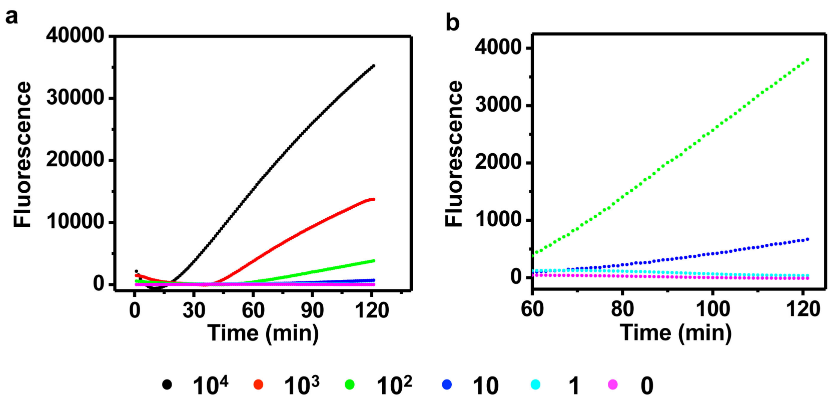

Supplementary Figure 9. *E. coli*-dependent HRCA reaction with human blood samples. Real-time monitoring of HRCA reactions with blood samples containing various concentrations *of E. coli* (cells mL^{-1}).

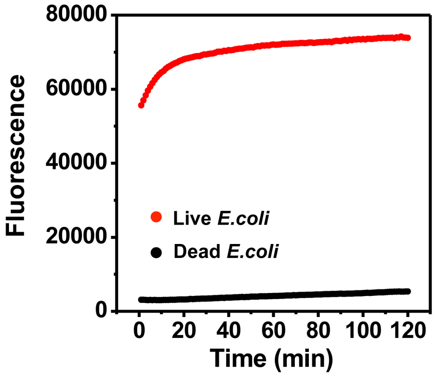

Supplementary Figure 10. HRCA based detection of live *E. coli* cells and dead *E. coli* cells (10^5 cells mL^{-1}). Freshly cultured *E. coli* cells were first centrifuged and re-suspended in 1× RB containing 2 mg mL^{-1} lysozyme. After being completely frozen at -20°C, the cell suspension was the incubated at 4°C for 5 h. The dead bacteria following this procedure and the same amount of live bacteria were used to prepare crude cellular mixtures, which were then used to induce the cleavage of rD2C1.

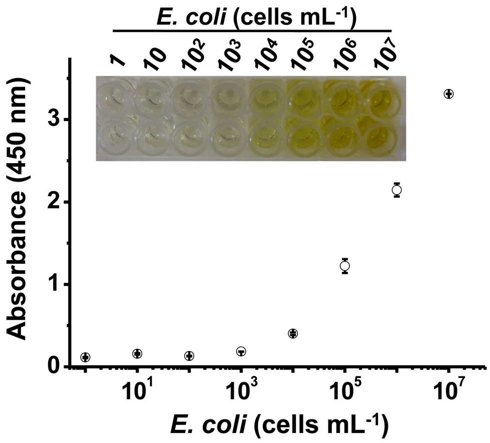

Supplementary Figure 11. Detection of *E. coli* using an ELISA kit. The detection is based on the binding of *E. coli* host cell protein to two antibodies, one immobilized on the microwells, and the other conjugated to horseradish peroxidase.

Supplementary Tables

Supplementary Table 1. Sequences of oligonucleotides used in this work (written in 5′-3′ direction)

**Synthesis of ^{C}DNA$_{i}$:** ^{C}DNA$_{i}$ is made via circularization of ^{L}DNA$_{i}$ in the presence of DNA$_{i}$CT as the template	
^{L}DNA$_{i}$	ACTGTAACCA TTCTTGTTTC GTATCATTGC AGAATTGATA TCATTTATCT GAATACCGTG
DNA$_{i}$CT	GTTACAGTCA CGGTAT
**Synthesis of ^{C}DNA$_{i}$CD:** ^{C}DNA$_{i}$CD is made via circularization of ^{L}DNA$_{i}$CD in the presence of DNA$_{i}$CT as the template	
^{L}DNA$_{i}$CD	ACTGTAACCA TTAAACCCAA CCCGCCCTAC CCAAAAGATA TCATTTATCT GAATACCGTG
**Synthesis of ^{C}DNA'$_{i}$:** ^{C}DNA'$_{i}$ is made via circularization of ^{L}DNA'$_{i}$ in the presence of DNA'$_{i}$CT as the template	
^{L}DNA'$_{i}$	TGCAGAATTG ATATCATTTA TAAAAAAAAA AAAACGGTGG CCATTCTTGT TTCGTATCAT
DNA'$_{i}$CT	AATTCTGCAA TGATACG
**Synthesis of r^{L}DNA$_{ii}$:** r^{L}DNA$_{ii}$ is produced via ligating FS28, DNA$_{ii}$F1 and DNA$_{ii}$F2 in the presence of DNA$_{ii}$T1 and DNA$_{ii}$T2 as ligation templates	
FS28	ACTCTTCCTA GCFRQGGTTC GATCAAGA
DNA$_{ii}$F1	CACGGTATTC AGTCTCTCTC TCTCTCTCT CTC
DNA$_{ii}$F2	TCTCTCTCTC TCTCTCTCTC AATGGTTAC AGT
DNA$_{ii}$T1	TAGGAAGAGT GAGAGAGA
DNA$_{ii}$T2	GAGAGAGAGA TCTTGATCG A
**Synthesis of r^{C}DNA$_{ii}$:** r^{C}DNA$_{ii}$ is made via circularization of r^{L}DNA$_{ii}$ in the presence of DNA$_{ii}$CT as the template	
DNA$_{ii}$CT	GAATACCGTG ACTGTAACC A
**Synthesis of r^{L}DNA'$_{ii}$:** r^{L}DNA'$_{ii}$ is produced via ligating FS28, DNA$_{ii}$F1 and DNA$_{ii}$F2' in the presence of DNA$_{ii}$T1 and DNA$_{ii}$T2 as ligation templates	
DNA$_{ii}$F2'	TCTCTCTCTC TCTCTCTCTC TTAGGCCAC CGT
**Synthesis of r^{C}DNA'$_{ii}$:** r^{C}DNA$_{ii}$ is made via circularization of r^{L}DNA'$_{ii}$ in the presence of DNA'$_{ii}$ CT as the template	
DNA'$_{ii}$ CT	GAATACCGTG ACGGTGGCCT
***E. coli*-responsive DNAzyme EC1 and its inactive mutant EC1M**	
EC1 (X = Inverted dT)	GATGTGCGTT GTCGAGACCT GCGACCGGAA CACTACACTG TGTGGGGATG GATTTCTTTA CAGTTGTGTG X
EC1M (X = Inverted dT)	GATGTGCGTA AAGCTCACCT GCGACCGGAA CACTACTGAC ACTGGGGATG GATTTCTTTA CAGTTGTGTG X
**DNA primers for RCA**	
DP1	AATTCTGCAA TGATACG
DP2	GCTAGGAAGA GTGAGA
**DNA primers for HRCA**	
FP1	GTTACAGTCA CGGTAT
RP1	CATTGCAGAA TTGATA
**DNA template for restriction digestion**	
DT1	CAGAATTGAT ATCATTTATCTG

Supplementary Table 2. Major advantages of our method in comparison with the traditional microbial detection system, a PCR test and ELISA for bacterium detection.

Method	Test time	Sensitivity
Roche Septifast[a]	**6.7 hours** (lysis 15 minutes; DNA extraction 90 minutes; DNA amplification 150 minutes; data analysis 30 minutes)	10-100 cells mL^{-1}
Biomerieux (BacT/ALERT® FA)[b]	**12.0-43.9 hours** (culture) for 10 cells per bottle or less; **10.8-35.2 hours** for 100 cells per bottle or less	10-100 cells mL^{-1}
ELISA[c]	**3 hours** (set-up 15 minutes; 1st incubation 60 min; 1st washing 10 min (5 washes); 2nd incubation 60 min; 2nd washing 10 min (5 washes); substrate incubation 15 min; stop step and data reading 10 min)	10^3 cells mL^{-1}
Our method	**2.3 hours** (sample preparation 18 minutes; DNAzyme 60 minutes; PNK/RCA 60 minutes)	10 cells mL^{-1}

[a]http://www.diagnomol.com/PDFs/Septifast.pdf

[b]http://www.biomerieux.co.kr/upload/Package_Insert_-_9304034-_B_-_en_-_259791[1]-2.pdf

[c]https://www.4adi.com/objects/catalog/product/extras/800-130-ECP.pdf

Supplementary Methods

Materials.

All DNA oligonucleotides (Supplementary Table 1) were purchased from Integrated DNA Technologies (IDT) and purified by 10% denaturing (7 M urea) polyacrylamide gel electrophoresis (dPAGE). T4 polynucleotide kinase (PNK), T4 DNA ligase and ϕ29 DNA polymerase (ϕ29DP) were purchased from Thermo Scientific. γ-[^{32}P]ATP was purchased from Perkin-Elmer. All other chemicals were purchased from Sigma-Aldrich and used without further purification. The autoradiogram images of gels were obtained using a Typhoon 9200 variable mode imager (GE healthcare) and analyzed using Image Quant software (Molecular Dynamics).

Preparation of ^{C}DNA$_{i}$.

Phosphorylation of ^{L}DNA$_{i}$. ^{L}DNA$_{i}$ was first labeled with γ-[^{32}P]ATP at the 5′ end using PNK according to the manufacturer's protocol. To ensure that all DNA molecules contained the 5′ phosphate required for the subsequent ligation reaction, PNK mediated end-labeling solution containing 5′-^{32}P labeled ^{L}DNA$_{i}$ was further incubated with 2 mM non-radioactive ATP at 37°C for 30 min. The phosphorylated DNA was purified by 10% dPAGE.

Circularization of ^{L}DNA$_{i}$. A total of 400 pmol of ^{L}DNA$_{i}$ was first mixed with 450 pmol DNA$_{i}$CT in 50 µL of H_2O, followed by heating at 90°C for 1 min. After cooling to room temperature and leaving the solution for 15 min, 10 µL of 10× T4 DNA ligase buffer (400 mM Tris-HCl, 100 mM $MgCl_2$, 100 mM DTT, 5 mM ATP, pH 7.8 at 25°C) and 10 U of T4 DNA ligase were added (total reaction volume was 100 µL) and the mixtures were incubated at room temperature for 2 h. ^{C}DNA$_{i}$ was concentrated by ethanol precipitation and purified by 10% dPAGE.

Preparation of ^{C}DNA$_{i}$CD.

^{C}DNA$_{i}$CD was prepared using similar procedures described for ^{C}DNA$_{i}$ except for the substitution of ^{L}DNA$_{i}$ with ^{L}DNA$_{i}$CD.

Preparation of r^{L}DNA$_{ii}$ and r^{C}DNA$_{ii}$.

Synthesis of r^{L}DNA$_{ii}$. r^{L}DNA$_{ii}$ was produced through T4 DNA ligase mediated ligation of FS28, DNA$_{ii}$F1 and DNA$_{ii}$F2 in the presence of DNA$_{ii}$T1 and DNA$_{ii}$T2 as ligation templates. A total of 400 pmol of FS28 was first mixed with 400 pmol of DNA$_{ii}$F2, 10 U of PNK and 5 mM ATP in 50 µL 1× PNK buffer (50 mM Tris-HCl, pH 7.6 at 25°C, 10 mM $MgCl_2$, 5 mM DTT, 0.1 mM spermidine). The mixture was incubated at 37°C for 1 h, followed by heating at 90°C for 1 min. Then 400 pmol of DNA$_{ii}$F1, 450 pmol of DNA$_{ii}$T1 and 450 pmol of DNA$_{ii}$T2 were added, heated at 90°C for 40 s, cooled down to room temperature and left for 10 min. To the above mixture were added 15 µL of 10× T4 DNA ligase buffer (400 mM Tris-HCl, 100 mM $MgCl_2$, 100 mM DTT, 5 mM ATP, pH 7.8 at 25°C) and 15 U of T4 DNA ligase, and the resultant mixture (total 150 µL) was incubated at room temperature for 2 h. The obtained r^{L}DNA$_{ii}$ was concentrated by standard ethanol precipitation and purified by 10% dPAGE.

Synthesis of r^{C}DNA$_{ii}$. A total of 300 pmol of r^{L}DNA$_{ii}$ was first phosphorylated using a similar protocol described above for the phosphorylation of ^{L}DNA$_{i}$. The phosphorylated r^{L}DNA$_{ii}$ was mixed with 400 pmol of DNA$_{ii}$CT in 50 µL of H_2O, heated to 90°C for 1 min, cooled to room temperature and left for 15 min. To this mixture were added 10 µL of 10× T4 DNA ligase buffer and 10 U of T4 DNA ligase, and the resultant mixture (total 100 µL) was incubated at room temperature for 2 h. The resultant r^{C}DNA$_{ii}$ was concentrated by ethanol precipitation and purified by 10% dPAGE.

Preparation of $r^LDNA'_{ii}$ and $r^CDNA'_{ii}$.

Synthesis of $r^LDNA'_{ii}$. $r^LDNA'_{ii}$ was prepared using similar procedures described for r^LDNA_{ii} except for the substitution of $DNA_{ii}F2$ with $DNA'_{ii}F2$.

Synthesis of $r^CDNA'_{ii}$. $r^CDNA'_{ii}$ was prepared using similar procedures described for r^CDNA_{ii} except for the substitution of r^LDNA_{ii} and $DNA_{ii}CT$ with $r^LDNA'_{ii}$ and $DNA'_{ii}CT$, respectively.

Preparation of rD2C1'

A total of 100 pmol of $^LDNA'_i$ was first phosphorylated by using PNK. Then 150 pmol of $r^CDNA'_{ii}$ was added and heated to 90°C for 30 s. After cooling to room temperature and leaving the solution for 15 min, 200 pmol of DNA'_iCT was added and allowed to react for 30 min. To the above mixture were added 15 μL of 10× T4 DNA ligase buffer and 15 U of T4 DNA ligase, and the resultant mixture (total 150 μL) was incubated at room temperature for 2 h. The obtained rD2C1' molecules were concentrated by standard ethanol precipitation and purified by 10% dPAGE.

Comparison of the Cleavage Activity of EC1 and EC1M in the Presence of *E. coli*.

E. coli K12 was grown onto a Luria Broth (LB) agar plate for 12 h at 37°C. A single colony was then taken and used to inoculate 2 mL of LB. After shaking at 37°C for 14 h at 250 rpm, the bacterial culture was serially diluted in 10-fold intervals. One *E. coli* glycerol stock containing an average of 5000 colony forming units (CFUs) per 100 μL was inoculated into 2 mL of LB and grown at 37°C for 6 h with shaking at 250 rpm. 1 mL of this culture was centrifuged at 13,000 g for 20 min at 4°C. The cell pellet was suspended in 200 μL of 1× RB (50 mM HEPES, 150 mM NaCl, 15 mM $MgCl_2$, pH 7.5). After being frozen at -20°C, *E. coli* cells were sonicated for 1 min and put on the ice for 5 min. This process was repeated three times. The cell suspension was then centrifuged at 13,000 g for 10 min at 4°C. The obtained supernatant was used as the CIM-EC for the experiment (CIM: Crude intracellular mixture; EC: *E. coli*).

The cleavage reaction with the CIM-EC was carried out by mixing 5 μL of CIM-EC, 1 μL of rD2C1 (5 μM), 4 μL of EC1 or EC1M (50 μM) and 10 μL of 2×RB. The above mixture was incubated at RT for 60 min, followed by 20% dPAGE analysis.

Degradation of rD2C1 by ϕ29DP in the Presence of EC1, *E. coli* and PNK.

A reaction mixture containing 5 μL of CIM-EC, 1 μL of rD2C1 (5 μM), 4 μL of EC1 (50 μM) and 10 μL of 2× RB was incubated at RT for 60 min. Then 1 μL of PNK (10 U $μL^{-1}$) was added and incubated at 37°C for 30 min. The digestion reaction was initiated by the addition of 1 μL of ϕ29DP (10 U $μL^{-1}$), 3 μL of 10× RCA reaction buffer and 5 μL of water. The reaction mixtures were incubated at 30°C for 30 min before heating at 90°C for 1 min, cooling to room temperature and 20% dPAGE analysis.

Cell Culture and miRNA Extraction.

The adherent breast cancer cell line MCF-7 was cultured in α-MEM media (GIBCO) supplemented with 10% fetal bovine serum (Invitrogen). These cells were cultured at 37 °C in a humidified atmosphere containing 5% CO_2. Small RNAs (<200 nt) were extracted and purified using the E.Z.N.A ®miRNA Kit according to the manufacturer's protocol including 1) cell lysis, 2) organic extraction, 3) large RNA removal. The RNA quantity was determined by measuring optical density at 260 nm using the NanoVue™ Plus spectrophotometer.

Restriction Digestion of RCA Products.

A 2 µL portion of the RCA products (Fig. 4a) was mixed with 4 µL of 100 µM DT1 and heated at 90°C for 5 min before cooled to RT and left for 20 min. This was followed by the addition of 1 µL of 10× Fast digestion buffer and 1 µL of FastDigest*Eco*RV. The reaction mixture was then incubated at 37°C for 10 min and analyzed by 10% dPAGE.

Effect of Blood on RCA Reactions.

One milliliter of the human blood sample was first centrifuged at 13,000 g for 10 min at 4°C to remove the plasma. After re-suspension in 1 mL of 50 mM HEPES buffer (containing 100 mM NaCl, 5 mM KCl, 1 mM $MgCl_2$ and 0.05% Tween 20), the sample was sonicated for 5 min to obtain blood cell suspension containing hemoglobin (a major PCR inhibitor). This sample was taken to set up five 50 µL RCA reactions made of 1 µL of $^{C}DNA_{i}$ (5 µM), 1 µL of DP1 (10 µM), 1 µL of ϕ29DP (10 U μL^{-1}), 1 µL of dNTPs (50 mM), 5 µL of 10× RCA reaction buffer, and 1, 2.5, 5, 10, and 25 µL of the blood cell suspension, supplemented with enough water to make up 50 µL. Each reaction was incubated at 37°C for 30 min.

Detection of *E. coli* in Blood Samples.

The whole human blood sample (Innovative Research) was drawn from a healthy person and treated by sodium citrate. Freshly cultured *E. coli* K12 cells were diluted and spiked in 1 mL of this blood sample. Then the sample was centrifuged at 13,000 g for 10 min at 4°C to remove the plasma. After washing twice with 50 mM HEPES buffer (containing 100 mM NaCl, 5 mM KCl, 1 mM $MgCl_2$ and 0.05% Tween 20), the cell pellet was suspended in 500 µL of 1× RB, sonicated for 1 min, put on the ice for 5 min, and sonicated for another 2 min. The cell suspension was then centrifuged at 13,000 g for 10 min at 4°C. 10 µL of the obtained supernatant was then mixed with 1 µL of rD2C1 (5 µM), 4 µL of EC1 (50 µM) and 5 µL of 4× RB was incubated at RT for 60 min. Then 1 µL of PNK (10 U μL^{-1}), 1 µL of ϕ29DP (10 U μL^{-1}), 1 µL of dNTPs (50 mM), 1 µL of FP1 (50 µM), 1 µL of RP1 (50 µM), 5 µL of 10× RCA reaction buffer, 2.5 µL of 20× EvaGreen and 17.5 µL of water and 22 µL of water were added. These reactions were carried out in BioRad CFX96 qPCR system set to a constant temperature of 30°C, and the fluorescence intensity was recorded in 1 min intervals.

Detection of *E. coli* Using an ELISA Kit.

CIM-EC samples from freshly cultured *E. coli* were prepared using similar procedures described above. The assay was performed according to the manufacturer's protocol.
